# Biomarkers in Thyroid Tumor Research: New Diagnostic Tools and Potential Targets of Molecular-Based Therapy

**DOI:** 10.4061/2011/631593

**Published:** 2012-02-16

**Authors:** Oliver Gimm, Maria Domenica Castellone, Cuong Hoang-Vu, Electron Kebebew

**Affiliations:** ^1^Department of Clinical and Experimental Medicine, Faculty of Health Sciences, Linköping University; Department of Surgery, County Council of Östergötland, 58185 Linköping, Sweden; ^2^CNR c/o Dipartimento di Biologia e Patologia Cellulare e Molecolare, Istituto di Oncologia Sperimentale “G. Salvatore”, Università Federico II, 80131 Napoli, Italy; ^3^Universitätsklinik und Poliklinik für Allgemein-, Viszeral- und Gefäßchirurgie, Martin-Luther Universität, 06097 Halle, Germany; ^4^Endocrine Oncology Section, Surgery Branch, National Cancer Institute, National Institutes of Health, Bethesda, MD 20892, USA

Thyroid cancer's incidence has increased dramatically over the last years and it now accounts for 2.6% of all new cancers when epithelial skin cancers are excluded. This increase has been partially attributed to improved screening methods, mainly ultrasonography identifying subclinical tumors. However, the evidence that also the number of more advanced tumors and thus thyroid-cancer-associated mortality is increasing suggests that the underlying cause is not completely understood [1]. The molecular basis of thyroid carcinogenesis has been widely investigated, leading to the discovery of oncogenes such as *BRAF*, *RAS*, and *RET* as major players in tumor development and progression. Recently, however, it has been shown that the rate of *BRAF* mutations that have been associated with a more aggressive tumor type in papillary thyroid carcinomas has increased over time [2]. This oncogenic addiction to specific genetic changes has highlighted thyroid cancer as an ideal model for targeted therapy using biological inhibitors and small molecule inhibitors of *RET* and *BRAF*, which have already reached the clinic [3]. Our ability to detect persistent and recurrent malignant thyroid tumors has also improved as a result of more sensitive biochemical tumor marker assays, although it is unclear whether these higher rates indicate clinically significant disease.

The articles published in this special issue shed new light on the molecular mechanisms involved in thyroid tumors and propose novel diagnostic and therapeutic targets against this disease. The results could have important clinical ramifications in the management of patients with thyroid tumors.

Thyroid follicular neoplasms represent a diagnostic challenge, as thyroid fine-needle aspiration biopsy cannot distinguish benign from malignant tumors in up to 30% of cases. An important goal would be to identify diagnostic and prognostic markers that could help avoid unnecessary surgeries, which can result in complications such as recurrent laryngeal nerve palsy and, in the case of bilateral thyroid surgery, hypoparathyroidism. The main preoperative diagnostic challenge concerns follicular thyroid lesions. Traditionally, the distinction between follicular thyroid adenomas and carcinomas is made histologically when there is evidence of capsular and/or vascular invasion. Preoperative markers are, therefore, sought after. Prabakaran and colleagues analyzed the expression levels of various genes in archival thyroid tissue. They found that *RAP2A*, a member of the ras family that is closely related to *Ras*, was significantly associated with higher expression in microdissected carcinoma cells that have invaded through the thyroid capsule and entered blood vessels than in thyroid tumor cells growing under the capsule. They concluded that *RAP2A *may be a biomarker associated with invasion of thyroid follicular cells. If their finding can be confirmed in larger studies, the evaluation of this marker in fine-needle aspiration aspirates may be warranted and informative.

In another study, T. Kobawala and colleagues report on the clinical utility of interleukin-8 (IL-8) and interferon-alpha in the diagnosis of thyroid diseases. IL-8 is a well-characterized chemotactic cytokine that is produced by macrophages and other cell types such as epithelial cells. Interferon-alpha is an antiviral and anti-proliferative agent that can stimulate both macrophages and natural killer cells to elicit an anti-viral response. It has also been shown to be active against tumors. The authors have analyzed 88 patients with various types of thyroid diseases. They found overall increased levels of serum IL-8 in patients with thyroid carcinoma as compared to various benign disorders. While there is a large overlap between the serum IL-8 levels in patients with malignant and benign thyroid diseases, these data support the idea that chronic inflammatory processes may play an important role in the development and progression of cancer and provide new therapeutic targets in malignant thyroid tumors. 

Among the novel diagnostic markers proposed in this issue, M. Hedayati and colleagues report on leptin levels in 83 patients with papillary thyroid and 90 healthy control persons. Leptin is a neuroendocrine hormone that has a variety of different effects including effects on the immune system and the thyroid gland. The authors found significantly higher serum leptin level in patients with thyroid cancer as compared to the control group including a difference by sex.

In another article, l. Giovanella and colleagues have investigated thyroglobulin as a marker of recurrent or persistent disease by analyzing the levels of thyroglobulin in lymph nodes in order to analyze the presence of metastatic thyroid tissue. Thyroglobulin is a protein synthesized by the thyroid gland and stored in the follicular lumen. Since, under normal conditions, thyroglobulin can only be found inside the thyroid gland, it has been used for decades as a sensitive tumor marker to detect persistent and recurrent disease in patients with differentiated thyroid cancer of follicular cell origin (the most common types of thyroid cancer). Thyroglobulin can, however, also be used preoperatively to guide the extent of surgery in patients with persistent or recurrent disease. They found that thyroglobulin levels higher than 1.1 ng/mL in aspirates from cervical lymph nodes were highly sensitive for the presence of metastases. Both sensitivity and specificity were higher than that of cytology from fine-needle aspirates. This approach can thus help guide the need and extent of lymph node dissection in patients who have suspicious lymph nodes on ultrasound but with inconclusive cytologic findings either in the preoperative setting or during follow up.

Finally, N. Burrows and colleagues report on the role of hypoxia-inducible factor-1 (HIF-1) in thyroid carcinoma aggressiveness. HIF-1 regulates the expression of several genes that have been shown to be involved in tumor cell survival, progression, metastasis, and even resistance to both chemotherapy and radiotherapy. In this study, the authors show that both hypoxia and oncogenic signaling pathways can induce HIF-1 in thyroid carcinoma. Based on their analysis, they also suggest that targeting HIF-1 might improve the poor therapeutic response of advanced thyroid carcinoma to radiotherapy. 

*Oliver Gimm* *Oliver Gimm* *Maria Domenica Castellone* *Maria Domenica Castellone* *Cuong Hoang-Vu* *Cuong Hoang-Vu* *Electron Kebebew* *Electron Kebebew*
